# Cellulose/TiO_2_ Humidity Sensor

**DOI:** 10.3390/s25051506

**Published:** 2025-02-28

**Authors:** Susana Devesa, Zohra Benzarti, Madalena Costa, Diogo Cavaleiro, Pedro Faia, Sandra Carvalho

**Affiliations:** 1 Centre for Mechanical Engineering, Materials and Processes (CEMMPRE), Advanced Production and Intelligent Systems (ARISE), Department of Mechanical Engineering, University of Coimbra, Rua Luís Reis Santos, 3030-788 Coimbra, Portugal; zohra.benzarti@dem.uc.pt (Z.B.); diogo.cavaleiro@dem.uc.pt (D.C.); sandra.carvalho@dem.uc.pt (S.C.); 2 Laboratory of Multifunctional Materials and Applications (LaMMA), Department of Physics, Faculty of Sciences of Sfax, University of Sfax, Soukra Road km 3.5, B.P. 1171, Sfax 3000, Tunisia; 3 Physics Department, University of Coimbra, Rua Larga, 3004-516 Coimbra, Portugal; madalenacosta@gmail.com; 4 Centre for Mechanical Engineering, Materials and Processes (CEMMPRE), Advanced Production and Intelligent Systems (ARISE), Electrical and Computer Engineering Department, FCTUC, University of Coimbra, Polo 2, Pinhal de Marrocos, 3030-290 Coimbra, Portugal; faia@deec.uc.pt

**Keywords:** cellulose, TiO_2_ nanoparticles, impedance spectroscopy, humidity sensor

## Abstract

Resistivity-type humidity sensors, which detect changes in electrical resistance in response to variations in environmental humidity, have garnered significant interest due to their widespread application in industry, agriculture, and daily life. These sensors rely on diverse materials for fabrication, but their increasing variety has contributed to the accumulation of electronic waste. As a biodegradable polymer, cellulose offers unique advantages, including a naturally hydrophilic structure and a large specific surface area. These properties enable cellulose to reduce e-waste generation while facilitating the efficient adsorption of water molecules. However, despite these benefits, humidity sensors based solely on cellulose often suffer from poor sensitivity due to its limited hydrophilicity and non-adjustable structure. To overcome these limitations, the development of composite materials emerges as a promising solution for enhancing the performance of cellulose-based humidity sensors. Combining the complementary properties of cellulose and TiO_2_, this work presents the development of a cellulose/TiO_2_ composite humidity sensor through a sustainable approach. The resulting composite material exhibits significantly improved sensitivity compared with a sensor fabricated purely from cellulose. To achieve this, TiO_2_ nanoparticles were incorporated into cellulose extracted from potato peels, and the composite film was fabricated using the casting method. The sensor’s performance was evaluated by analyzing the dependence of its complex impedance, measured over a frequency range between 2 kHz and 10 MHz, while varying relative humidity (RH).

## 1. Introduction

Humidity sensors are widely used in modern society for applications such as real-time environmental monitoring, smart agriculture, and industrial processes including logistics, packaging, and storage [1,2]. Based on their sensing mechanism, humidity sensors are categorized into various types, including resistive, capacitive, surface acoustic wave, and quartz crystal microbalance sensors [3,4]. Compared with other types of sensors, capacitive humidity sensors have been considered by many researchers as the first choice for humidity detection due to their advantages of easy fabrication, low power consumption, small size, high sensitivity, quasi-linear response, and low-temperature drift [4]. Their working principle is based on capacitance changes caused by moisture-induced changes in the effective dielectric constant of ceramic or polymer dielectrics. Quartz crystal microbalances (QCMs), which everyday are attracting more attention, are extremely stable devices capable of measuring nanogram mass changes [5]. The working principle of QCM-based humidity sensors relies on the resonant frequency changes caused by changes in the surface quality after the hygroscopic material absorbs water [4]. QCM humidity sensors have the advantages of a low detection limit, wide measurement range, fast response speed, and long life, with their biggest limitation being the measuring range, which is up to around 55% at room temperature [4].

Resistive-type sensors keep gathering significant attention due to their simple fabrication method, small size, cost-effectiveness, and suitable circuit integration [1,4,6]. These sensors operate by detecting changes in the resistance caused by interactions between the sensing material and water molecules. Their operating principle relies on the resistance change caused by the adsorption of water molecules on the sensitive membrane. The benefit of a resistive sensor is its high surface–volume ratio, which allows it to measure humidity changes in the environment up to 90% relative humidity at room temperature.

Recent advancements in wearable electronics and robotics have increased the demand for flexible humidity sensors, as they allow for easy mounting on curved surfaces such as human skin and robotic limbs, seamless integration with other functional modules, and enhanced robustness to mechanical stress. Consequently, the development of flexible electronic devices has broadened the application scope of humidity sensors [1].

To create flexible sensors, a variety of substrates have been investigated, including polyimide (PI), polydimethylsiloxane (PDMS), polyester (PE), and polyethylene terephthalate (PET) [2]. However, advancements in science and technology are increasingly shifting towards the use of eco-friendly materials, renewable resources, and sustainable methods and processes [7].

Over the past several decades, natural polymers have gained significant attention for their role in creating biocompatible and environmentally sustainable materials. Among these, cellulose stands out as the most abundant renewable polymer resource. Its renewability, biodegradability, biocompatibility, and strong affinity to water due to its numerous hydroxyl groups make it an excellent candidate for use in flexible humidity sensors [8]. Despite its advantages, traditional cellulose-based humidity sensors face limitations in achieving a wide detection range and high sensitivity, which significantly restricts their application [9,10]. Recent studies indicate that the moisture sensitivity of cellulose-based humidity sensors can be enhanced by incorporating hydrophilic substances into the cellulose matrix [9].

Among these substances, titanium dioxide (TiO_2_), due to its excellent hydrophilicity, chemical stability, affordability, and relative sensitivity to humidity, has been used in the preparation of humidity sensors [10,11]. TiO_2_ has a bandgap width of 3.2 eV and presents excellent conductivity, high surface activity, and a high sensitivity to oxygen content [12]. In the past, flexible humidity sensors based on TiO_2_ and cellulose derivatives, such as ethyl cellulose [13] or cellulose nanocrystals [14], have already been explored; however, a comprehensive understanding of their humidity-sensing mechanisms, as analyzed through electrical impedance spectroscopy measurements, remains relatively unexplored, presenting significant potential for further development and refinement (electrical impedance spectroscopy (EIS) provides information about both the materials’ bulk and their inner and outer interfaces, and can be used to assess how charges are bounded or transferred through the systems and/or the existing interfaces).

This is the case in the work by Tong et al. [15], which fabricated hybrid TiO_2_/cellulose films for humidity sensing, where their best performant composite exhibited a high humidity response, rapid response/recovery speed, and high stability. However, they do not present a discussion about humidity mechanisms based on EIS evaluation.

Therefore, in the present work, which intends to attenuate the production of e-waste and to enhance moisture sensitivity, a humidity sensor made from cellulose extracted from potato peels, impregnated with TiO_2_ nanoparticles, was fabricated through a sustainable, cost-effective process. It should be reinforced that the proposed approach will specifically allow for the reduction of e-waste [16], because it has been shown that the inclusion of TiO_2_ nanoparticles promotes photo- and thermo-degradation of biopolymeric materials [17] The fabricated composites were evaluated by means of electrical impedance spectroscopy, which allows the identification of the mechanisms contributing to the overall observed conductivity, and this provides support to future materials composition design with an upgraded response.

The performance of the developed sensor was evaluated by measuring its complex impedance under an externally applied sinusoidal electric field, while varying the relative humidity. The obtained impedance data permitted the identification of the humidity-sensing mechanisms contributing to the overall electrical response observed. An equivalent electrical circuit was established within a specific frequency range to achieve a good fit with the experimental data, offering adequate physical interpretation based on well-established mechanisms described in the literature.

## 2. Materials and Methods

### 2.1. Samples Preparation

Potato peel waste consists of starch, cellulose, hemicellulose, lignin, and other impurities, and therefore several pretreatments are required, comprising alkali treatment and chlorite bleaching, to isolate its cellulosic component [18,19].

The potatoes, purchased at a farmers’ market, were hand-peeled, and the removed peel was thoroughly washed with water to remove any remaining soil and other contaminants.

A mixture of deionized water and potato skins, with a water-to-pulp weight ratio of 20:1, was then blended for 10 min to remove most of the potato flesh. Afterwards, the prepared slurry was filtered using a 100 μm sieve and then washed with deionized water. Subsequently, the peel waste was treated with a 0.5 mol/dm^3^ aqueous sodium hydroxide solution (NaOH, 98%, Panreac, Barcelona, Spain) at 70–80 °C for 2.5 h under mechanical agitation.

This alkali treatment procedure was performed three times to ensure the complete elimination of lignin, hemicellulose, and other undesired impurities; at the end of each treatment, the pulp was filtered with a 45 μm sieve and rinsed with deionized water to remove soluble impurities.

Finally, the remaining solid material was bleached at 60–70 °C for 2 h using a sodium chlorite aqueous solution (NaClO_2_, 80%, Thermo Scientific, Waltham, MA, USA) containing an acetate buffer solution (2.3 wt%), composed of sodium acetate (CH_3_COONa, ExpertQ^®^, Scharlab, Barcelona, Spain) and acetic acid (CH_3_COOH, ≥99.8%, Merck, Rahway, NJ, USA). The pH of the final product was adjusted to 4.9 using hydrochloric acid (HCl, 37% *v*/*v*, Panreac). This process was performed four times, to assure the elimination of organic impurities.

To prepare the cellulose/TiO_2_ films, 0.05 wt% of TiO_2_ nanoparticles (>99.5%, Nanografi, Ankara, Turkey, with an average size of 45.0 nm) were incorporated into the cellulose slurry and stirred until homogeneity was achieved. The prepared mixture was transferred into a polystyrene Petri dish, where it was pressed and then freeze-dried. Subsequently, the Petri dish cover was taken off, and the resulting films were air-dried, revealing a 1.74 wt% content of TiO_2_ nanoparticles. A control sample was created following the same procedure, but without the addition of TiO_2_ nanoparticles.

The samples were named as follows: cellulose film, for the control, and cellulose/TiO_2_ film, for the composite.

### 2.2. Characterization Techniques

The X-ray diffraction (XRD) analysis of the cellulose film was performed using a Pontypool, Wales, UK Rigaku Smartlab diffractometer, Tokyo, Japan, with Cu Kα (λ = 1.54060 Å) radiation, operating at 40 kV and 50 mA. The tests utilized a Bragg–Brentano geometry, with a step size of 0.04° and an exposure time of 4.8 s per step. The TiO_2_ nanoparticles were analyzed on a PANalytical X’Pert PRO diffractometer, Almelo, The Netherlands, with Cu Kα (λ = 1.54060 Å) radiation, operating at 45 kV and 40 mA. The tests utilized parallel beam geometry, with a step size of 0.04° and an exposure time of 3 s per step.

Raman spectroscopy measurements were carried with a Renishaw inVia™, Wotton-under-Edge, UK, confocal Raman spectrometer, employing a 532 nm green laser (power of 50 mW).

The surface of the films was examined using scanning electron microscopy (SEM) on a SU3800 microscope from Hitachi, Tokyo, Japan, in secondary electron mode. The chemical composition of the films was analyzed via EDS with a Bruker Nano system, set at an accelerating voltage of 10 keV.

Electrical impedance spectroscopy (EIS) measurements were executed using an Agilent 4294A, Santa Clara, CA, USA, in the frequency range of 2 kHz–10 MHz to evaluate the electrical response of the samples to moisture changes (the applied excitation signal was a sinusoidal shaped one possessing a maximum amplitude of 0.5 V and 0 V bias).

**Figure 1:**
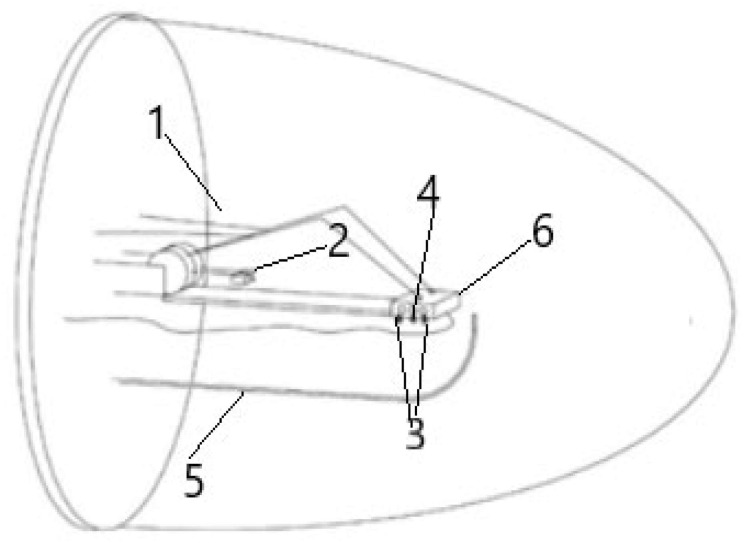
Measuring chamber schematics (1—electrodes, 2—counterweights, 3—heating elements, 4—thermocouple, 5—g inlet and outlet, 6—samples holder).

The measurements were always performed under constant air flow by placing the samples in a support equipped with measuring contacts inside a closed chamber which possess a volume of approximately 6.5 L (see Figure 1). The different values of RH were obtained by mixing, in the chosen ratio, water-saturated air, obtained by bubbling synthetic air through water in a bubbler, with dry synthetic air. The volumetric flow rates of both saturated wet and dry air were controlled by independent mass flow controllers (Teledyne Hastings Instruments HFM-200 Thermal mass flow controller/meter, Hampton, VA, USA) and then joined together in a helicoidal mixer before passing tangentially over the surface of the sensors placed inside the test chamber. The RH percentage concentrations were checked and validated by means of the gas chromatography technique using a gas chromatograph, GC (GC1000, DANI, Mylan, Italy). The temperature inside the chamber was electronically controlled with an accuracy greater than 1 °C and maintained at 25 °C (ambient temperature). The temperature was measured by means of a type K thermocouple, inserted in the same support where the heating elements were fixed (the support has already been mentioned, and samples were placed for electrical measurements), with the control performed using a GEFRAN controller model 1000 running the Proportional Integrative Derivative (PID) algorithm.

Once the chamber inlet had been placed in a way that the flow passed tangentially over the sample’s surface, an air flow of 5 L/h was chosen; this decision was based on assuring a reasonably fast change of the chamber’s content (the chamber possessed a volume of around 6.5 L, so to ensure a full complete mixture renovation inside it, a 90 min waiting period was adequate when considering the ratio mentioned), and simultaneously to reduce fluctuations to the maximum sensor measuring temperature (because air flow can impact the surface temperature of materials, even when considering that below the sample was a measure/control heating system).

For performing sample evaluation (for which two acquisition runs were performed), gold electrodes were applied to the surface extremities of each sample using gold ink from the Gwent Group (Gold Polymer Electrode Paste C2041206P2), Pontypool, Wales, UK, cured at 30 °C for one week. The data integrity of the measured complex impedance was ensured by the Agilent equipment, which included inbuilt Kramer–Krönig transformations [20].

**Figure 2:**
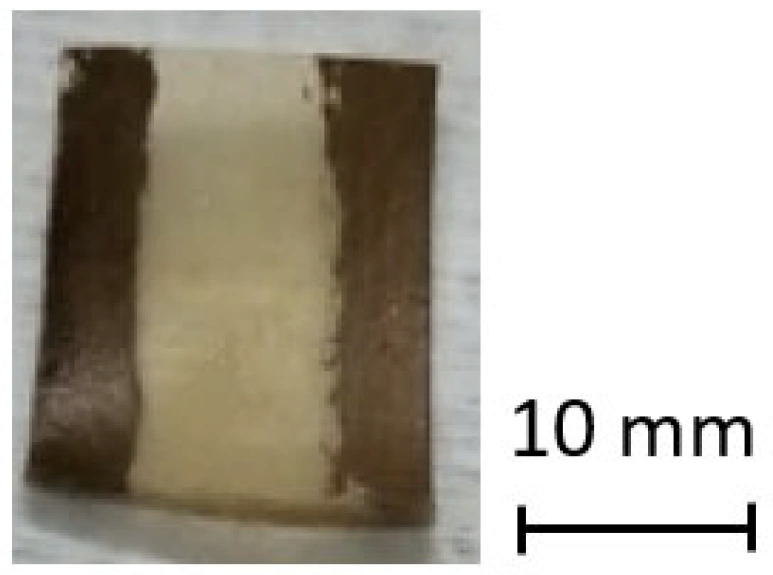
Photograph of the fabricated sensor sample.

In Figure 2, a photograph of the sensors produced and tested is presented. As mentioned before, a pair of electrodes was placed on the top surface of the sensor for performing the electrical assessment. Then, it was placed in the sample holder inside the chamber, and the measuring contacts were elastically pressed against the electrodes (see the chamber description in Figure 1). The sensors’ dimensions are approximately 10 × 12 mm length and width, respectively, with each electrode possessing a width of 2.4 mm.

## 3. Results

**Figure 3:**
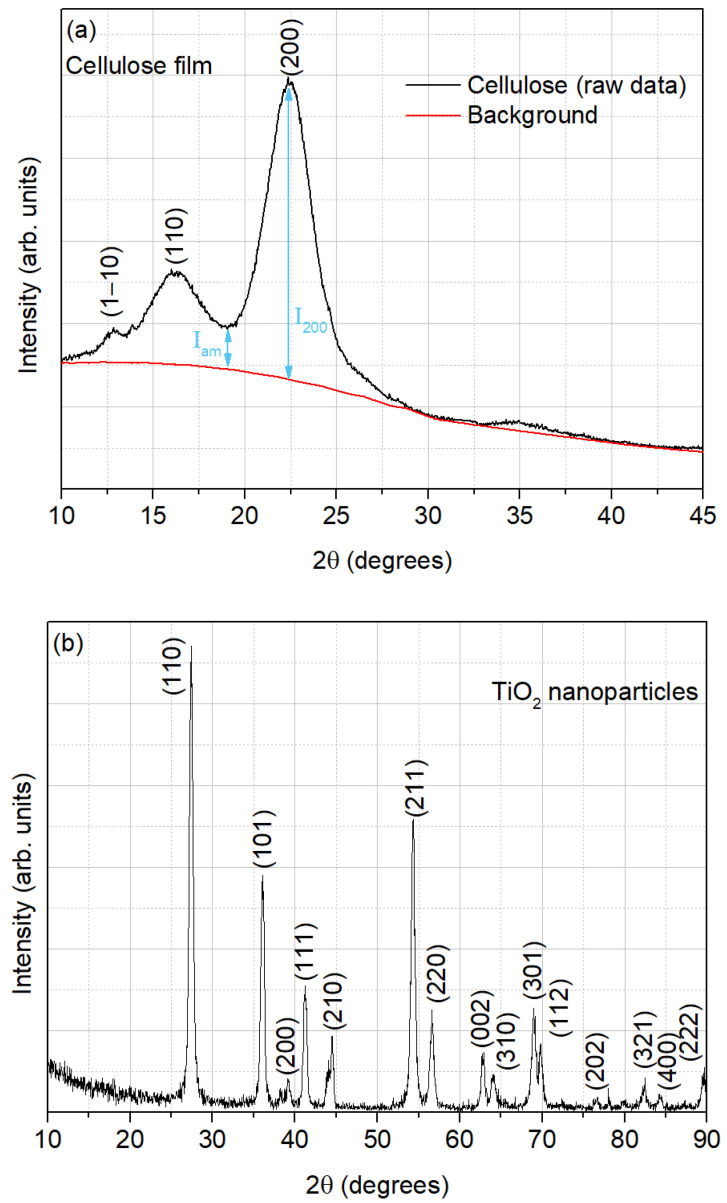
Diffractogram of (**a**) cellulose film and (**b**) TiO_2_ nanoparticles.

The XRD pattern of the film from the extracted cellulose is presented in Figure 3a, where three diffraction peaks, centered around 12.8°, 16.2°, and 22.5°, can be observed. This result is in accordance with the characteristic diffraction peaks of cellulose I [21].

The crystallinity index (CrI), which expresses the relative degree of crystallinity, was calculated according to the Segal method [22], as depicted in Equation (1). This method, also known as the peak height method, is an empirical method that is the most common and simple method to determine the degree of crystallinity in native cellulose [23]:

(1) $$CrI\left(\%\right)=\frac{I_{200}-I_{am}}{I_{200}}\times 100\%$$

where I_200_ is the maximum intensity (in arbitrary units) of the 200 lattice diffraction (crystalline region) and I_am_ is the intensity of diffraction, in the same units, of the non-crystalline material (total intensity after the subtraction of the background signal), which is taken at an angle of about 18–19° in the valley between the peaks [22,23], as also shown in Figure 3a.

From Equation (1), the estimated crystallinity index for the cellulose extracted from potato peels was 85.77%. This high degree of crystallinity can be attributed to the removal of lignin and hemicellulose in amorphous regions during the alkali treatment, and to the elimination of organic impurities through the bleaching process [24].

The crystallinity index of the cellulose produced in this study is comparable with that calculated for the index extracted from durian peel waste (81.62%) [24] using ammonium persulfate oxidation. However, it is higher than indices reported in studies employing the same oxidation method but using different cellulose sources, such as oil palm fronds (52.4%) [25], balsa (57.65%), kapok fibers (60.71%) [26], recycled medium density fiberboard (62.7%) [27], Pennisetum Purpureum fibers (66.56%) [28], *Miscanthus x. Giganteus* (70%) [29], and sugar cane bagasse pulp (76.5%) [30]. In comparison, the crystallinity index for cellulose extracted from cotton linters has been reported as 93.5% [31], higher than that estimated for this work on cellulose, although the procedure used in the present research has the advantage of not relying on the use of ammonium persulfate. Ammonium persulfate is known as being a strong oxidizer with low long-term toxicity, high water solubility, and low cost [32]; however, its utilization results in the formation of two major byproducts—sulfuric acid and ammonium sulfate [33]. If not properly recovered, these byproducts can lead to significant environmental impacts, particularly in terms of soil acidification and water eutrophication [34].

The higher crystallinity in cellulose contributes to unique properties such as a high aspect ratio, large surface area, excellent mechanical strength, high thermal stability, and an optically active surface, among others. These characteristics make it suitable for the development of composites, films, and barriers, as well as for various applications, particularly in sensing-related fields [35,36,37].

The XRD patterns of the TiO_2_ nanoparticles, as illustrated in Figure 3b, were analyzed and compared with the reference data provided in the standard International Centre for Diffraction Data (ICDD) file 04-003-0648 [38]. The observed diffraction peaks correspond to the ones displayed by the rutile polymorph of TiO_2_, indicating a good agreement with the standard data.

**Figure 4:**
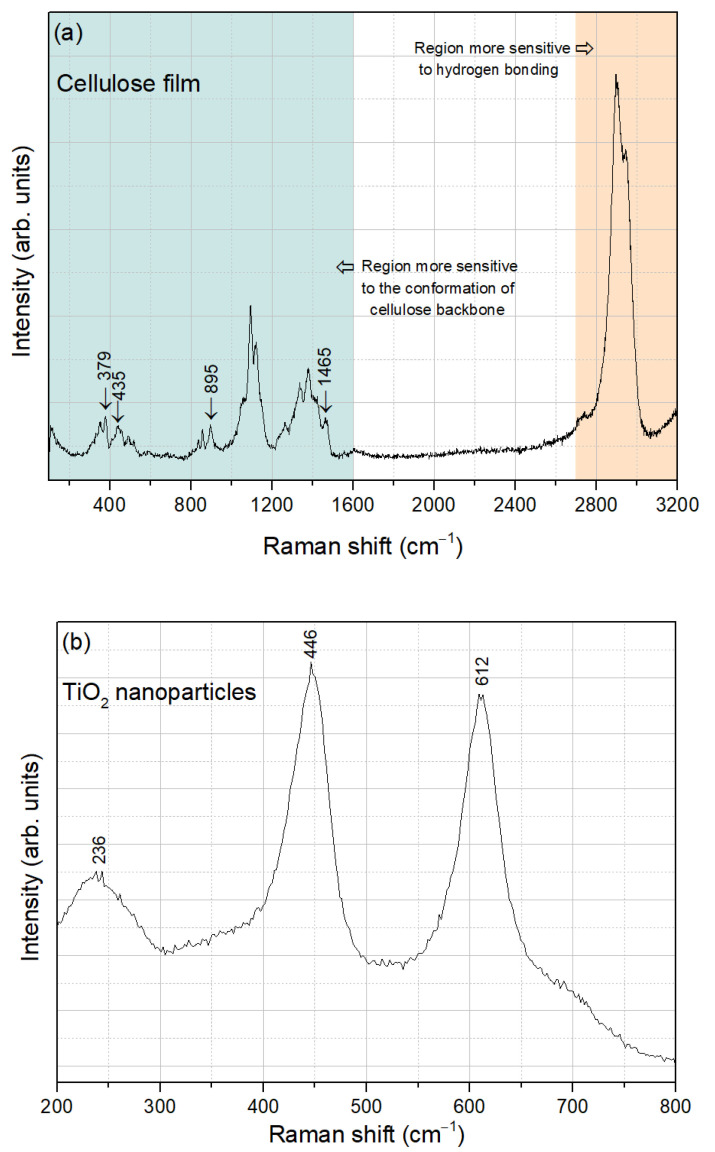
Raman spectra of (**a**) cellulose film and (**b**) TiO_2_ nanoparticles.

Figure 4a presents the Raman spectrum of the extracted cellulose film, recorded in the spectral range of 100–3200 cm^−1^. This spectrum can be divided into two regions: the region below 1600 cm^−1^, which is most sensitive to the conformation of the cellulose backbone, and the region above 2700 cm^−1^, which is more sensitive to hydrogen bonding [39]. The Raman bands characteristic of cellulose I, indicated by the arrows, include those centered at 379 cm^−1^ and 435 cm^−1^, which are attributed to CCC, COC, OCC, and OCO skeletal bending, to CCH and COH methylene bending, and to the movement of CC and CO groups within the glucopyranose ring units. Additionally, the band at 895 cm^−1^ corresponds to HCC and HCO bending, while the band at 1465 cm^−1^ is related to HCH scissoring bending [40].

Regarding the TiO_2_ nanoparticles, the Raman spectrum obtained in the spectral range of 200–800 cm^−1^ is shown in Figure 4b. The bands centered at 236 cm^−1^, 446 cm^−1^, and 612 cm^−1^ are characteristic of rutile TiO_2_ [41], demonstrating good agreement with the XRD analysis.

**Figure 5:**
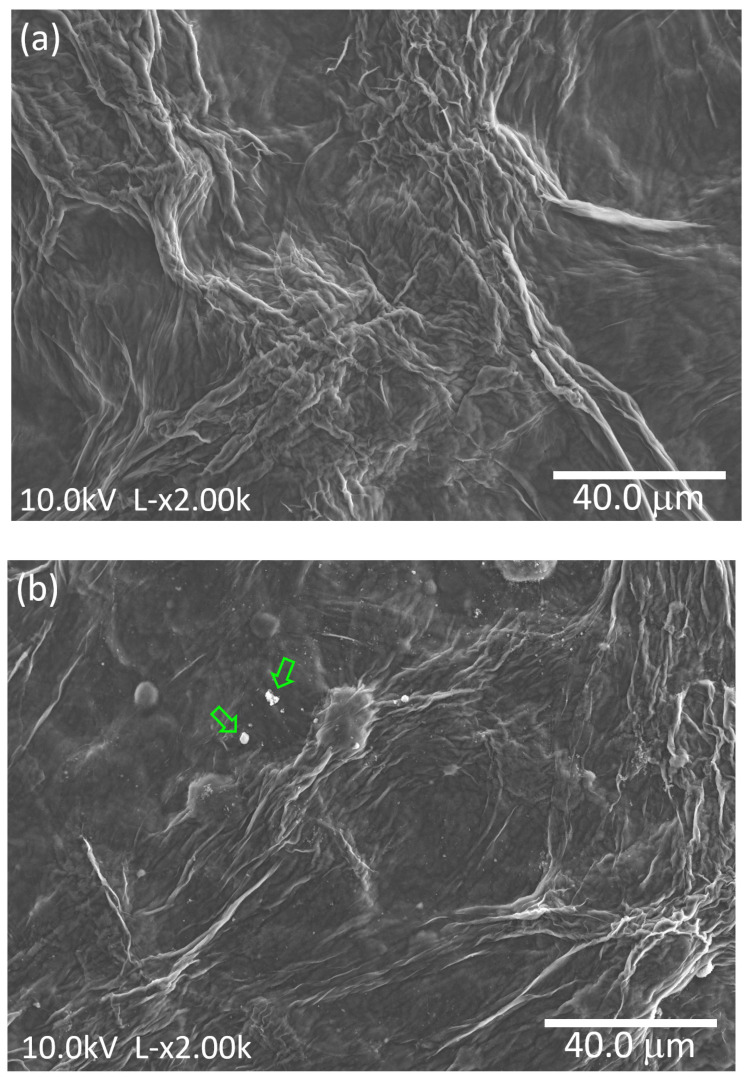
SEM micrographs of (**a**) cellulose and (**b**) cellulose/TiO_2_ films.

Figure 5 shows the SEM micrographs of cellulose film, as well as the micrographs of the cellulose film with incorporated TiO_2_ nanoparticles, the cellulose/TiO_2_ film.

The cellulose film, illustrated in Figure 5a, displays a dense structure with no apparent porosity and consists of fibers with varying diameters, ranging from a few tens of nanometers to hundreds of nanometers. Similarly, Figure 5b reveals comparable features, suggesting that the stirring process employed to integrate the TiO_2_ nanoparticles did not substantially alter the morphology of the cellulose film. Furthermore, the nanoparticles are visibly present, with the formation of some aggregates (indicated by arrows).

**Figure 6:**
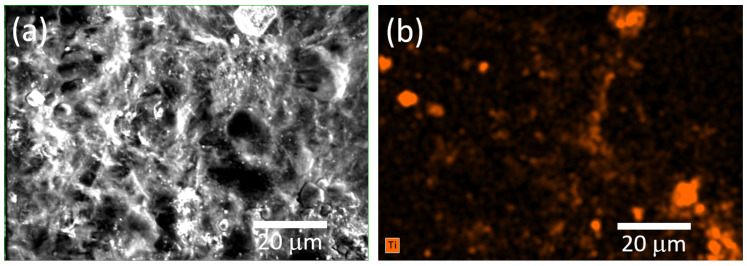
(**a**) SEM micrograph and (**b**) corresponding EDS elemental mapping of the cellulose/TiO_2_ film.

Figure 6 displays the elemental mapping of Ti within the cellulose/TiO_2_ film, showing that the TiO_2_ nanoparticles are dispersed throughout the cellulose matrix. Nevertheless, the presence of clusters, as previously mentioned, is evident. Studies have shown that TiO_2_ nanoparticles in suspensions tend to aggregate naturally, a phenomenon commonly observed in such systems. Even when advanced dispersion techniques or extended sonication procedures are employed, most nanoparticles still form large clusters or aggregates. This behavior, widely reported for nano-TiO_2_ and other metal oxide nanoparticles, is consistent with colloidal chemistry principles. Key factors such as ionic strength and pH in aqueous environments play a crucial role in influencing this aggregation [42,43].

Thus far, the majority of the existing resistive/impedance humidity sensors display a decrease in the electrical resistance as the humidity concentration increases [44]. The mechanism of cellulose aligns with the traditional impedance-type humidity sensor [2]. In this case, the dominant conduction mechanism is of ionic nature, with the impedance of the sensor decreasing with increasing RH due to the physisorption and capillary condensation of water molecules on the material’s surface [45].

**Figure 7:**
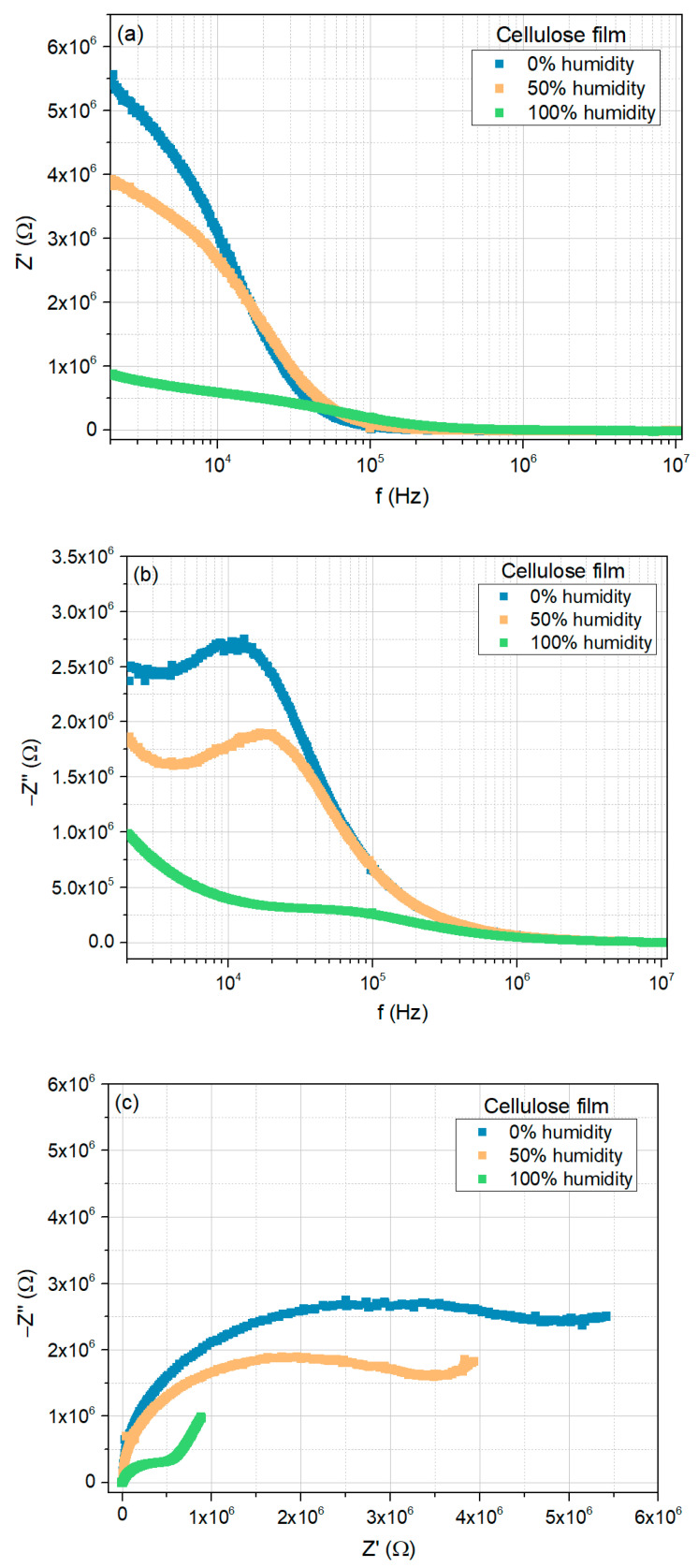
Electrical response of the cellulose film to moisture (0%, 50%, and 100%), at room temperature: (**a**) real part of the impedance as a function of frequency; (**b**) imaginary part of the impedance as a function of frequency; (**c**) Nyquist plots.

Figure 7a,b show the real and imaginary parts of the impedance Z′ and Z″, respectively, measured at room temperature, as a function of frequency, for the cellulose film, when exposed to 0%, 50%, and 100% relative humidity concentrations.

In the low-frequency region, the real part of the impedance (Figure 7a) decreases with the increase in RH. However, as the frequency increases, the curves tend to merge, displaying a trend that is nearly independent of both frequency and RH concentration.

The imaginary part of the impedance (Figure 7b) also exhibits a similar trend for higher frequencies. However, instead of displaying a monotonic decrease in the low-frequency region, a peak appears, shifting to higher frequencies as RH increases. This peak indicates the presence of a relaxation mechanism within the frequency range under study.

Figure 7c shows the corresponding Nyquist plots. In a 0% humidity environment, the Nyquist plot exhibits a circular arc with a large radius; nevertheless, what seems to be a straight line is visible in the low-frequency region. With the increase of the RH concentration, this line segment becomes clearer while the radius of the semi-circular arc decreases. Thus, although the dominant contribution to the electrical response arises from the grain, the contribution from the diffusion mechanisms increases with increasing moisture content [45].

**Figure 8:**
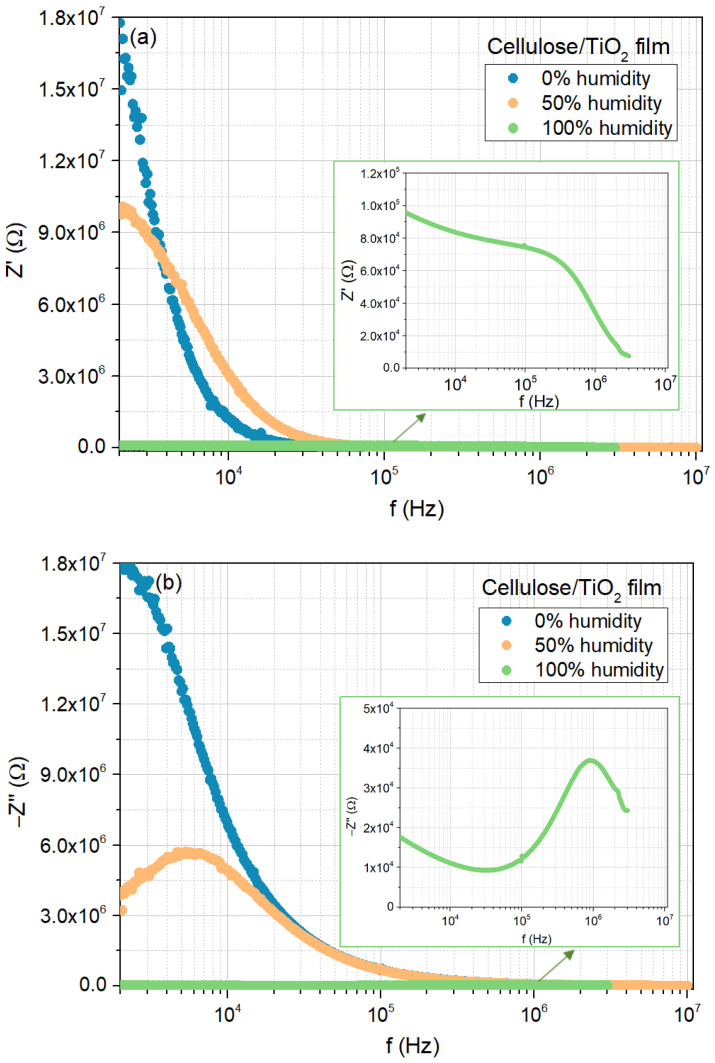

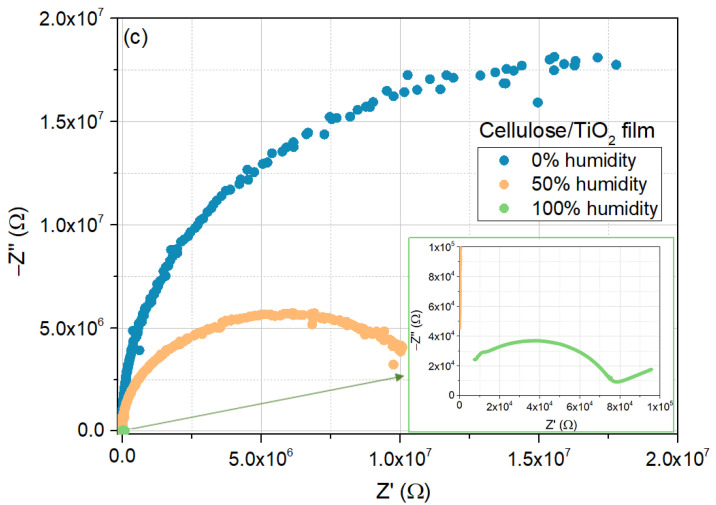
Electrical response of the cellulose/TiO_2_ film to moisture (0%, 50%, and 100%), at room temperature: (**a**) real part of the impedance as a function of frequency; (**b**) imaginary part of the impedance as a function of frequency; (**c**) Nyquist plots.

The same analysis was conducted for the cellulose/TiO_2_ film, as shown in Figure 8a–c, which exhibited similar behavior. The major differences can be found in the magnitude of the real and imaginary parts of the impedance in the low-frequency region, as well as in the lack of evidence of the contribution from the diffusion mechanisms to the electrical response for the 0 and 50% moisture concentrations.

**Figure 9:**
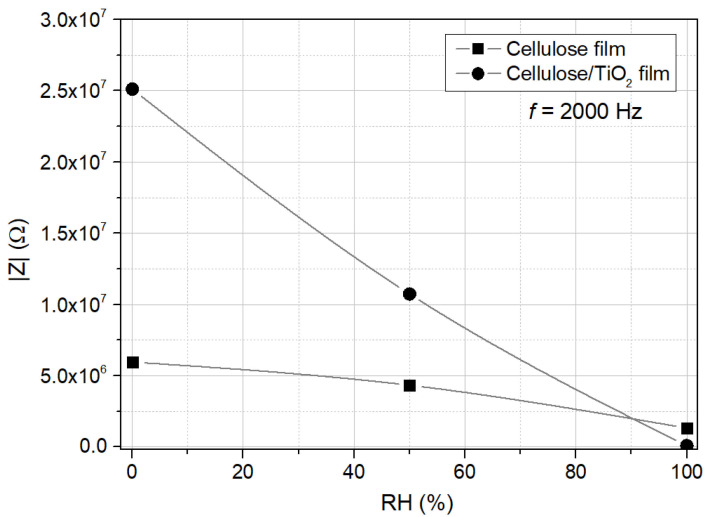
Electrical response of cellulose and cellulose/TiO_2_ films to moisture (0%, 50%, and 100%); impedance modulus, measured at room temperature and 2000 Hz.

Figure 9 compares the electrical response to humidity of the cellulose and cellulose/TiO_2_ films, showing the impedance modulus obtained at room temperature and 2000 Hz. The variation of the impedance modulus with relative humidity (RH) confirms the negative humidity impedance behavior for both films. At 2 kHz, the impedance modulus decreases from 5.97 MΩ (RH 0%) to 4.33 MΩ (RH 50%) and then to 1.32 MΩ (RH 100%) for the cellulose film, and from 24.11 MΩ (RH 0%) to 10.73 MΩ (RH 50%) and then to 0.10 MΩ (RH 100%) for the cellulose/TiO_2_ film. This represents a total decrease of 4.5 times for the cellulose film and 258.3 times for the cellulose/TiO_2_ film (more than two orders of magnitude variation), demonstrating that TiO_2_ nanoparticles can be incorporated into a cellulose matrix to enhance its humidity-sensing properties. The estimated sensitivity, S, of the fabricated cellulose/TiO_2_ film was around 241 for RH = 100% (the sensitivity was calculated using the impedance modulus obtained at 2 kHz, by the expression S=Z0RRH, where $\left|Z_{0}\right|$ and $\left|R_{RH}\right|$ stand for the impedance modulus for RH = 0% and for another under exposure RH, respectively).

As it is confirmed that electrical resistance decreases as humidity concentration increases, the basic humidity-sensing mechanism of the cellulose film can be inferred. Due to the high content of hydroxyl groups in cellulose, the film readily adsorbs moisture from the air, leading to a reduction in the material’s resistance [46]. Initially, during the diffusion of free volume, water molecules form weak interactions with cellulose because of the hydrophilic nature of its hydroxyl groups. This chemical adsorption process creates a monolayer of water molecules. Once the first layer is formed, and moisture concentration continues to grow, physical adsorption replaces chemisorption, where two adjacent hydroxyl groups interact with corresponding water molecules. During physisorption, as higher humidity levels are reached, additional layers of water molecules are subsequently formed. This process aligns with the Grotthuss chain reaction mechanism [42], resulting in a gradual capillary condensation of water molecules, as described by Kelvin’s equation [10,47,48].

For the case of TiO_2_, the humidity-sensing mechanism is like that typically observed for metal oxide-based sensors and can be explained in two steps: (i) a chemisorption process at low humidity levels, followed by (ii) a physisorption process at medium/high humidity levels, accompanied also by the Grotthuss chain reaction [49].

At lower RH percentages, the chemisorption of water vapor takes place on the sensing material’s surface sites. These sites consist primarily of defects, such as oxygen vacancies and metal interstitials; they exhibit a high local charge density and a strong electrostatic field, which originate the dissociation of water molecules into protons (H^+^) and hydroxyl groups (OH^−^). The water molecules are then chemisorbed onto the sensor’s surface. Proton hopping between hydroxyl groups contributes to increased conduction. This chemisorbed layer remains unaffected by changes in humidity [50,51].

At higher RH concentrations, the physisorption of water molecules takes place over chemisorption; the physiosorbed layers are formed continuously on top of each other and of the chemisorbed layer. During this step, water molecules combine with protons to form H_3_O^+^ species. These H_3_O^+^ ions release protons to neighboring water molecules, triggering a chain reaction. This process, the Grotthuss chain reaction, leads to increased ionic conduction due to proton hopping as RH keeps rising [50,51,52].

Since the TiO_2_ nanoparticles are generally well dispersed within the cellulose matrix, the just-described combined mechanisms for both cellulose and metal oxide explain the enhanced humidity-sensing properties of the cellulose/TiO_2_ film.

Given the promising results of the cellulose/TiO_2_ film, its electrical response to humidity was investigated in greater detail.

**Figure 10:**
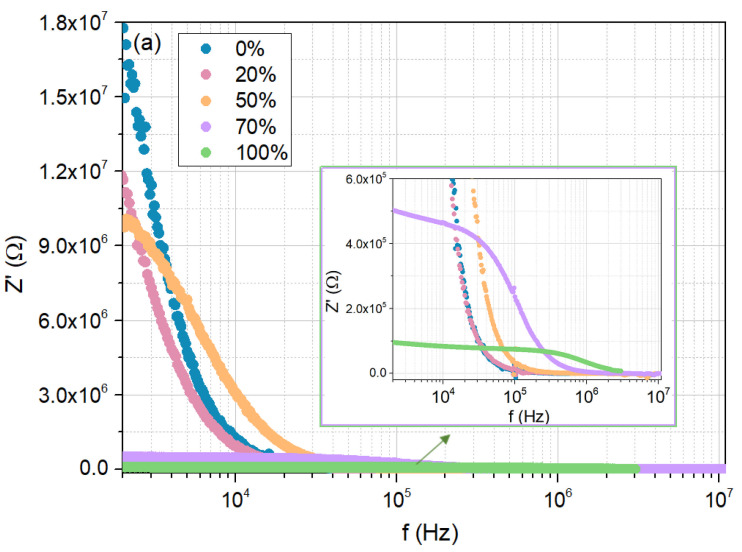

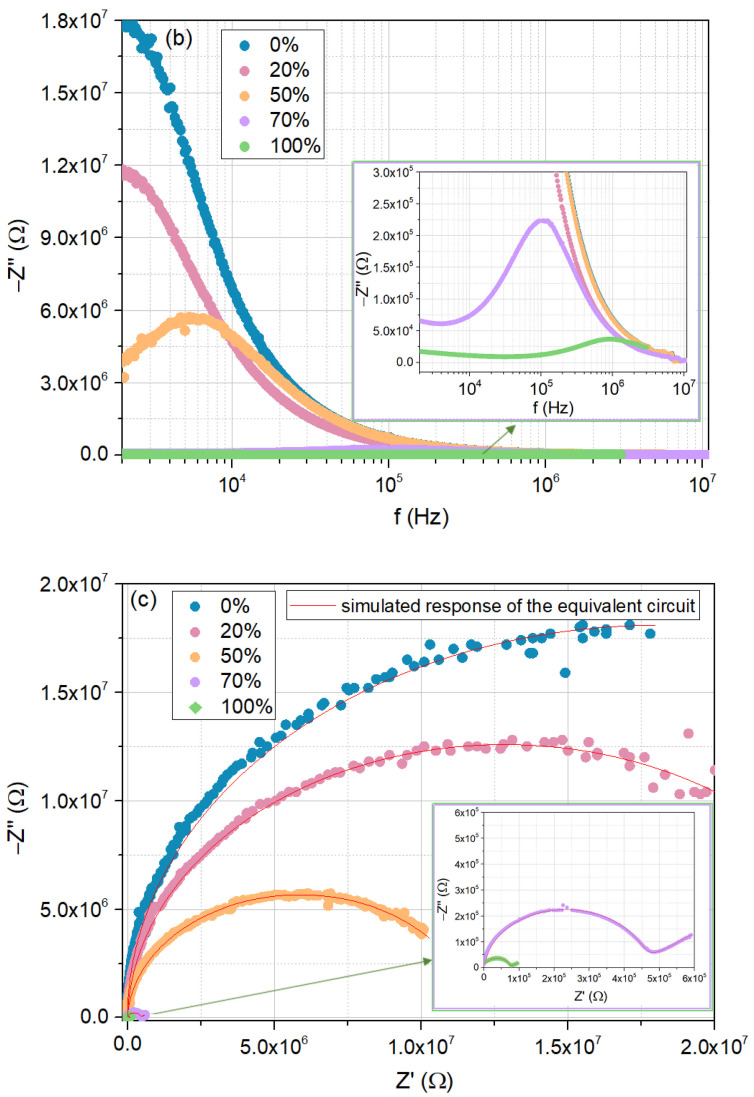
Electrical response of the cellulose/TiO_2_ film to moisture (0%, 20%, 50%, 70%, and 100%), at room temperature: (**a**) real part of the impedance as a function of frequency; (**b**) imaginary part of the impedance as a function of frequency; (**c**) Nyquist plots.

Figure 10a,b show the real and imaginary parts of the impedance Z′ and Z″, respectively, measured at room temperature, as a function of frequency, for the cellulose film, but now when exposed to a 0%, 20%, 50%, 70%, and 100% moisture concentrations.

The results obtained for 20% and 70% humidity conditions, concerning the evolution of Z′ and Z″ with frequency, are consistent with previous measurements, confirming the already discussed trends. Specifically, in the low-frequency region, both Z′ and Z″ decrease with increasing RH, while the peak representing the relaxation mechanism shifts to higher frequencies as RH increases.

Regarding the corresponding Nyquist plots (Figure 10c), under 20% RH, a circular arc is noted, similar to the tendencies found for 0% and 50% RH. With the increase in humidity to 70%, a “tail” emerges, indicating the contribution of diffusion mechanisms, as seen for 100% RH.

Different equivalent circuits that match humidity sensors’ behavior with moisture, representing its effect on the electrical conduction and polarization of the materials, are proposed and found in the literature [53]. Often, a single data set can be reasonably fitted by multiple equivalent circuits, making the choice dependent on both simplicity and consistency with the known physical and chemical processes occurring in the system [45].

In this study, the impedance data were modeled by means of two equivalent circuits, with the model circuits’ parameters having been obtained using the EIS Spectrum Analyzer fitting Software, version 1.0 tool [54]. The simulated response of the equivalent circuit is also shown in Figure 10c.

The first equivalent circuit, which reflects the dominant influence of the grain at lower relative humidity values (0% to 50%), consists of a resistance R_1_ in parallel with a constant phase element CPE_1_ (in the frequency domain, the impedance of a constant phase element is Z=Af−ncosnπ2−jsinnπ2, where A is the impedance observed for the angular velocity of 1 rad/s, *f* is the frequency, and n is an exponent, equal to 1 for a the case of a pure capacitor). CPE_1_ was used to replace capacitance and account for non-ideal Debye behavior [55]. For higher relative humidity values (70% and 100%), a diffusion component was included, represented by a second constant phase element CPE_2_ in series with the initial circuit.

**Figure 11:**
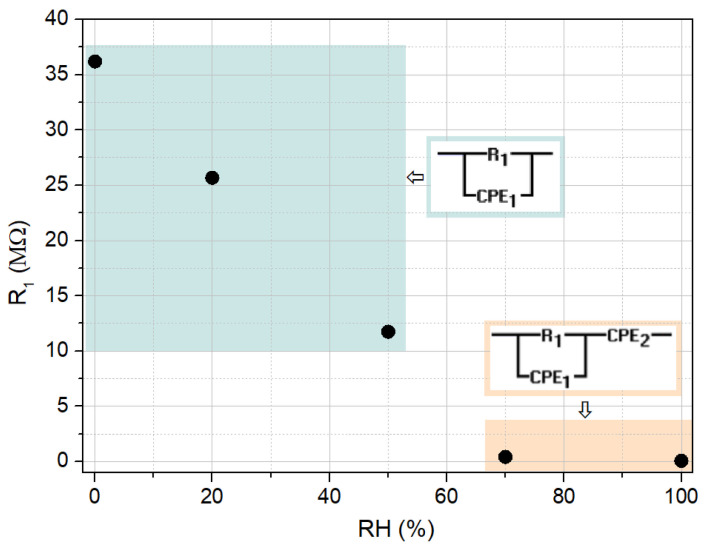
Dependence of the grain resistance on relative humidity and corresponding equivalent circuits adopted.

In Figure 11, the model circuits used to evaluate the impedance data and the dependence of the grain resistance on relative humidity are illustrated. Table 1 summarizes the estimated circuit parameters values obtained by fitting the experimental data using the mentioned simulation tool.

The obtained parameters complement the discussion regarding the conduction mechanisms concerning the electrical response found of the cellulose/TiO_2_ film with moisture. Indeed, the grain resistance contribution decreases, initially caused by the superficial adsorption of water molecules and later also due to their capillary condensation along the cellulose/TiO_2_ film structure. Additionally, for higher moisture concentrations, once ionic conduction contribution increases, its corresponding circuit element CPE_2_ admittance (generally, Q=1A), also displays a significant increase, as it rises almost four times higher when the RH concentration rises from 70 to 100%.

Lastly, in Table 2 a brief comparison between the present study’s results and similar cellulose/TiO_2_-based compositions is reported. The estimated sensitivity for the present work’s cellulose/TiO_2_ film was around 241 for RH = 100%, which is only surpassed by the cellulose nanocrystal of eucalyptus pulp fiber/TiO_2_ film reported by Wu et al. [14], which displayed a sensitivity of around 450. However, their film did not exhibit linear behavior in the range of 0–100%; its output displayed linear variation in the RH range of 11–54% and an almost linear change in the remaining evaluated RH range of 54–95%, but the adjusted slopes of the two regions are quite different. In any case, the film here reported can assess RH concentration in the entire range of RH of 0–100%, displaying an almost linear electrical response; to the best of the authors’ knowledge, none of the remaining films are capable of a similar result.

## 4. Conclusions

The cellulose/TiO_2_ composite film developed in this paper demonstrates significant potential as a high-performance humidity sensor due to its enhanced measuring range, sensitivity (S = 241 for RH = 100%), and stability, which combined are better than those listed for similar composites in the literature. Structural characterization confirmed the high crystallinity index (81.08%) of cellulose extracted from potato peels, attributed to the effective removal of amorphous impurities during processing. This value surpasses most reported indices for cellulose from alternative sources, underscoring the sustainability and efficiency of the extraction method used.

The uniform dispersion of TiO_2_ nanoparticles within the cellulose matrix, albeit with some aggregation, was evident from SEM and EDS analyses.

Impedance spectroscopy evaluation revealed a pronounced negative humidity impedance behavior, with the cellulose/TiO_2_ film exhibiting a decrease in impedance by a factor of 258.3 across the relative humidity range, compared with a decrease by a factor of 4.5 for pure cellulose. The combined effects of chemisorption and physisorption facilitated by TiO_2_ provide a comprehensive explanation for the enhanced ionic conduction observed.

The impedance data fitted using equivalent model circuits highlighted the dominant grain contributions at lower humidity levels and the increasing role of diffusion mechanisms at higher humidity levels. The fitting parameters, such as grain resistance, align with the expected trend, decreasing resistance with increasing humidity, and further validate the proposed mechanisms.

In conclusion, this cellulose/TiO_2_ composite film, developed through an eco-friendly process utilizing food waste, offers a robust and sustainable solution for humidity sensing. Its performance surpasses that of pure cellulose-based sensors, and exceeds the majority of those found in published written works for similar composites, providing a viable pathway for advancing bio-friendly and efficient sensor technologies.

## Figures and Tables

**Table 1:** Fitted results for the grain resistance, R_1_, and admittances Q_1_ and Q_2_ of the CPE elements of the developed sensor.

RH (%)	R_1_ (MΩ)	Q_1_ (pS)	n_1_ (a.u.)	Q_2_ (μS)	n_2_ (a.u.)
0	36.18	2.21	1.00	_―_	_―_
20	25.68	3.86	0.987	_―_	_―_
50	11.74	3.08	0.976	_―_	_―_
70	0.43	3.73	0.998	0.20	0.427
100	0.07	2.62	0.997	0.74	0.396

**Table 2:** Comparative analysis for similar composites (sensitivity was estimated by S=Z0RRH, by S=R0RRH, or by S=CRHC0, depending on the type of operating principle).

Sensing Material	Mechanism/Sensitivity	Response Type	Reference
Potato peel cellulose + TiO_2_ nanoparticles	Resistive/around 241 (for RH = 100%)	Almost linear in the range 0–100%	Present work
Ethyl Cellulose + TiO_2_	Capacitive/6 (for RH = 96%)	Linear in the range 11–85% RH	[13]
Cellulose nanocrystal of eucalyptus pulp fiber + TiO_2_	Resistive/around 450 (for RH = 95%)	Linear in the range 11–54% and almost linear in the range 54–95%	[14]
Cellulose nanocrystals of cotton pulp + TiO_2_	Resistive/around 1900 (for RH = 97%)	Almost linear in the range 54–97%	[15]

## Data Availability

Data will be made available on request.
